# Impact of the Deionized Water on Making High Aspect Ratio Holes in the Inconel 718 Alloy with the Use of Electrical Discharge Drilling

**DOI:** 10.3390/ma13061476

**Published:** 2020-03-24

**Authors:** Magdalena Machno, Rafał Bogucki, Maciej Szkoda, Wojciech Bizoń

**Affiliations:** 1 Institute of Rail Vehicles, Faculty of Mechanical, Cracow University of Technology, 31-155 Cracow, Poland; maciej.szkoda@pk.edu.pl; 2 Institute of Materials Engineering, Faculty of Materials Engineering and Physics, Cracow University of Technology, 31-155 Cracow, Poland; rbogucki@mech.pk.edu.pl; 3 Institute of Production Engineering, Faculty of Mechanical, Cracow University of Technology, 31-155 Cracow, Poland; wojciech.bizon@pk.edu.pl

**Keywords:** difficult-to-cut material, Inconel 718 alloy, micro-drilling, EDM, aspect ratio hole, deionized water

## Abstract

Nickel-based superalloys are being increasingly applied to manufacture components in the aviation industry. The materials are classified as difficult-to-machine using conventional methods. Nowadays, manufacturing techniques are needed to drill high aspect ratio holes of above 20:1 (depth-to-diameter ratio) in these materials. One of the most effective methods of making high-aspect-ratio holes is electrical discharge drilling (EDD). While drilling high aspect ratio holes, a crucial issue is the flushing of the gap area and the evacuation of the erosion products. The use of deionized water as the dielectric fluid in the EDD offers a considerable potential. This paper includes an analysis of the influence of the machining parameters (pulse time, current amplitude and discharge voltage) on the process performance (drilling speed, linear tool wear, taper angle, hole’s aspect ratio, side gap thickness), during the EDD with the use of deionized water in the Inconel 718 alloy. The obtained through holes were subjected to the extended analysis. The impact of the initial working fluid temperature and pressure on the conditions of the flow through the electrode channel was also subjected to the analysis. The deionized water properties were changed by applying an initial temperature. Based on the results of an analysis of the previous research, the EDD of the through holes was performed for a pre-set initial temperature (~313.15 °K) and initial pressure of the working fluid (8 MPa) and selected process parameters. An analysis of the results indicates increasing of hole’s aspect ratio by about 15% (above 30), decreasing the side gap thickness by about 40% and enhanced surface integrity.

## 1. Introduction

Nickel-based superalloys, such as Inconel 718, play an increasingly important role in the development and manufacture of aircraft engines components (such as turbine blades, guide vanes). The industrial use of Inconel 718 alloy started in 1965; hence, it is a relatively recent alloy. The Inconel 718 alloy is characterized by excellent mechanical properties, excellent resistance to creep at temperatures up to 973 °K, and corrosion and oxidation resistance in aggressive environments [1]. 

The major alloyed composition of the material includes Ni and Cr. Additionally, in the chemical composition, elements such as Al, Ti, Nb, Co, Cu and W occur. Fe can also be added in amounts ranging from 1% to 20% [1]. The alloy elements such as Ni and Cr provide the corrosion resistance of the Inconel 718 and crystallize as a γ phase (the precipitation fine hard and dispersed precipitates, i.e., *γ′* and *γ″*). The added Ni element forms hardening precipitates *γ″* (Ni3Nb, a body centered tetragonal metastable phase). On the other hand, Ti and Al participate in order to form of intermetallic *γ′* (Ni3 (Ti, Al), simple cubic crystal). The added C element participates in the forming of MC carbides (M = Ti or Nb), but the C content must be low enough to enable Nb and Ti precipitation in the form of *γ′* and *γ″* particles. In addition, Mo is often the content element of the material increasing the mechanical resistance by solid solution hardening [1,2]. In order to obtain high mechanical properties, the alloy is subjected to heat treatment. It involves annealing in the temperature range of 1273–1473 °K for 1 hour with cooling in water with subsequent aging in the temperature range from 923 to 1173 °K in order to separate the coherent phases *γ′* and *γ″,* which are responsible for high hardness and alloy strength [3]. Too low annealing temperature may lead to the formation of undesirable NbC, δ-Ni3Nb and Laves phases, resulting in reduced plasticity, fatigue and creep properties [4]. The high hardness of the alloy (average hardness 414 Hv) combined with the low thermal conductivity (11.4 W/(m∙°K)) is the cause of problems during machining [5].

Due to extremely tough nature (such as lower thermal conductivity, high work hardening, presence of abrasive carbide particles, high hardness, affinity to react with tool material, high toughness) of the Inconel 718 superalloy, the making of high aspect ratio holes in its structure involves significant difficulties in the case of conventional machining [5,6]. In order to overcome the limitations, the aerospace industry currently applies non-conventional methods such as electrochemical machining (ECM), laser beam machining (LBM) and electrical discharge machining (EDM) to produce micro-scale cooling holes in superalloy materials [7]. 

In modern gas turbine engines, in order to improve the turbine’s operating efficiency, a high temperature of the gas before the turbine (in the range of 823.15–1373.15 °K) is applied, which decreases the components’ resistance. To enhance the additional resistance of these materials of components into high temperature, a considerable number of holes (20,000–40,000) with a diameter in the range of 0.3–5 mm and an aspect ratio in the range of (40–600):1 (depth-to-diameter ratio) are made in their structure [5,8,9,10]. The main task of the holes (named “cooling holes”) is to reduce the temperature of the component material by flow of the cooling factor (gas or liquid) through the holes [7]. The efficiency of the cooling process depends on the dimensional shape accuracy and the quality of the holes’ inner surface. One of the most effective methods of drilling high aspect ratio holes in the nickel-based superalloys is electrical discharge drilling (EDD) [11,12].

### 1.1. Electrical Discharge Drilling Process (EDD)

In the EDD process, the allowance is removed through electrical discharges that occur between two electrodes (one of the electrodes being the workpiece and the other one the tool) in a narrow gap (~µm) filled with the working fluid, and the forces occurring between the tool and the workpiece surface are negligible or do not occur. The transformation of electrical energy into thermal energy leads to the vaporization and melting of the material of the workpiece and of the tool electrode [13,14,15]. Due to the presence of high temperature (about 10,000 °K) in the machining zone, the quality of the machined surface is unsatisfactory because of the occurrence of erosion micro-craters, re-solidified material, heat affected zone and white layer (recast layer) involving micro-cracks and residual tensile stresses [16,17,18]. A disadvantage of the process is also a low material removal rate and high tool wear reaching above 50% [19]. However, the process of electrical discharge drilling offers the possibility of making burr-free holes with high-precision (accuracy of <5 μm) in a range of materials regardless of their hardness as long as the material is electrically conductive. Typical drilled hole diameters are in the range of 0.008–0.5 mm and are characterized by a depth-to-diameter ratio of 20:1 or higher [19,20,21]. 

In the electrical discharge process, the materials usually selected for making tool electrodes are copper, bronze, zinc, tungsten, graphite. The chosen tool electrode should provide criteria such as being good conductor, high electrode resistance to wear, machinability, enough surface roughness. To provide these criteria, the commonly materials to make tool electrode are copper and graphite [22,23]. In the case of electrical discharge drilling of deep holes in Inconel 718, a copper tube-electrode is more appropriate providing high material removal rate and low surface roughness [24]. The tool electrode made of copper is characterized by a high electrical conductivity (up to 60.9 MS/m) and a high thermal conductivity (388 W/(m∙°K)), which are the properties needed in materials used to make tool electrodes in the EDD process. However, when deionized water (consisting of hydrogen and oxygen) is used as working fluid, the conditions of high temperature at the hole bottom can contribute to form a passive layer on the surface of the cooper tool electrode and machined surface of the Inconel 718 alloy. In addition, hydrogen penetrating the structure of copper material reduces the amount of oxides. On the other hand, water vapor forming into the copper structure influences high pressure, which can cause cracks in the material structure. In the case of the workpiece, the content of Cr in the Inconel 718 alloy structure should provide the oxidation resistance at high temperature conditions [25].

In the EDD process, the making of high aspect ratio holes is a challenge due to the difficulties involved in removing the process products such as debris and gas bubbles from the gap area (especially if the drilling hole is deep) [26,27,28]. Consequently, the debris accumulates at the hole bottom leading to the occurrence of abnormal/secondary discharges (such as arc and/or short circuits) between the debris and the sidewall of the hole. In the result, a poor surface quality (increased roughness parameters *Ra* and *Rz*), decreased hole accuracy and machining speed, excessive tool wear, decreased gap area occur [14,26,27,29,30]. Abnormal discharges can also occur in the corner of the hole bottom. In [31], the analysis of experimental research shows that the crack density is higher at the edge of the hole bottom, which may attest to the occurrence of secondary discharges in the zone. Secondary discharges also cause an excessive wear of the electrode’s tip edge [21,32,33,34].

In addition, due to secondary discharges, unremoved and re-melted debris starts to attach and accumulate on the electrode’s surface. Then, electrical discharges can occur between the re-solidified debris on the electrode’s surface and the hole wall. In [21], the measurements of the tool electrode after drilling show an increase in the electrode’s diameter by about 38 μm. This can affect the decrease of the side gap thickness and contribute to difficulties of the working fluid flowing outside of the hole thus decreasing the process stability. In [35], the authors also analyze the attachment of the debris to the electrode. The attachment of the debris takes place in the central region of the electrode’s tip, with a smaller amount at the tip’s edge. It suggests that the mechanism of attachment involves re-melting of the debris and does not occur randomly from the dielectric.

Previous papers concerning the experimental research into the EDD process emphasize the significance of efficient flushing of the gap zone [20,32,36]. Generally, a crucial role in the EDD process is played by the working fluid as a flushing agent, which cools the material of both electrodes (also remelted debris) and removes the machining particles from the discharge gap. However, efficient flushing often requires additional fixtures or adjustment of the machine. It is worth underlining that the properties of the working fluid such as electric conductivity and viscosity can substantially affect the working fluid flow, machining efficiency, electrode wear and recast layer thickness [18,37].

### 1.2. The Working Fluid Flow through the Electrode Channel and the Interelectrode Gap

The process efficiency depends strongly on the removal of the eroded particles, which is highly influenced by the flushing and thus the rate of the flow of the working fluid out of the hole. Flushing affects the quality of the drilled holes (dimensional and shape accuracy, low roughness parameters of the inside of the hole’s surface), process performance, electrode tool wear. In [38,39], the authors highlighted the fundamental role of the flushing system during the drilling of holes. Efficient gap zone flushing constitutes the main factor ensuring stability of the EDD process, especially in the case of drilling high aspect ratio holes. 

The flushing efficiency can depend on the length and the inside shape (single-channel, multi-channel) of the electrode tool [40] and the working fluid’s initial pressure [38]. The dielectric fluid’s pressure affects the increase in the metal removal rate (MRR) and the reduction of the surface roughness. In [14], the analysis of the research results shows that the optimum volumetric flow rate can ensure a constant interelectrode gap, which can decrease the number of secondary discharges. The application of a high-volumetric flow rate (25 l/h) decreases the electrode tool wear due to appropriate cooling of the electrode material and sufficient removal of the debris from the gap. The electrode’s diameter can also affect the kind of flow conditions in the electrode channel and the gap area. For the outer diameter of 0.5 mm of the tube-electrode, the laminar flow conditions in circular tube are considered. With the diameter of 1.0 mm, turbulent flow of the working fluid can take place. In addition, an analysis of the results proves that the simple cylindrical flushing channel inside of the electrode provides the best flow performance. The single-channel electrode provides comparatively better removal rates of the erosion products and a lower electrode wear ratio than multi-channel electrodes.

To analyze the flushing, the simulations were also performed. In [41], a mathematical model was developed that considers tool movement in a solid–liquid two-phase gap flow field and a 3D model to simulate the tool movement and debris generation. The analysis of the results of the model developed shows that with an increase in the flushing velocity, the fluid at the bottom collects more debris. In addition, the fluid flow is limited. The inner diameters of tube (single- or multi-channel) electrodes with a diameter of under 1.0 mm are too small to ensure effective gap flushing.

There are several flushing methods such as internal, external, suction-assisted flushing, flushing with different electrode movements or vibration- supported flushing [36]. However, external flushing can contribute to the vibration of a thin and long electrode [21].

### 1.3. Significance of the Working Fluid in the EDM Process

In electrical discharge machining, the application of deionized water as dielectric fluid indicates considerable potential since it can be treated as slightly conductive electrolyte, and electrodischarge erosion is accompanied by electrochemical dissolution [16,42]. The reaction of electrochemical dissolutions is possible due to resistivity of deionized water ranging widely between 0.1 and 10 MΩ cm based on its purity [43].

The EDM with the use of deionized water is often termed as a hybrid process of simultaneous Electrical Discharge and Electrochemical Machining (SEDCM) or Electrochemical Discharge Machining (ECDM). A major advantage of the process is the removal of the material by simultaneous interaction of electrochemical dissolution and electrical discharges in a single impulse [44]. The electrochemical dissolution improves the process performance (the maximum material removal rate in deionized water can be about 2.5 to 3 times faster) and reduces the tool electrode wear (the tool wear can be reduced up to 96%), in comparison to the application of oil-based dielectric [45]. The electrochemical reaction also enhances the quality of the machined surface by removing erosion micro-craters and re-solidified material on the rim [16,42]. After the EDM with the use of deionized water, micro-parts with a surface roughness parameter of *Ra* = 22 nm [16] and *Ra* = 12–43 nm were obtained [43]. Additionally, an analysis of the results of the EDM with the use of deionized water in the Inconel 718 alloy shows that the recast layer thickness can be reduced to the average thickness of 3–8 μm [18]. Overall, for machining efficiency and machined surface quality, deionized water is a better dielectric for electrical discharge machining of nickel-based superalloys.

In the case of reinforced electrochemical dissolution, the machined surface can be damaged, and the dimensional and shape accuracy of the hole can decrease [16,42,43,44,45,46]. In [45], the authors analyze the influence of deionized water on the dimensional accuracy of the machined parts. When applying low resistivity (of about 0.1 MΩ cm), the machined micro-column is tapered due to excessive electrochemical dissolution. The increase in resistivity of up to 12 MΩ cm reduces the tapered shaped of the column. This is because the high resistivity of water reduces the discharging distance and suppressed electrochemical dissolution. In addition, as the resistivity of deionized water decreases, the amount of oxidized materials increases. The oxidized material is experimentally proven to be nonconductive, and hence, there is no effect on the machining process. The similar results were determined in [47]. On the other hand, in [42], the high-frequency bipolar pulse generator that is applied offers the possibility of drilling micro-holes in deionized water without electrolytic corrosion of the surface near the hole top. In addition, the hole’s inner surface is improved (surface roughness *Ra* = 0.105 µm) due to a small amount of electrochemical dissolution.

The material chemical composition of the surface after the EDM process using deionized water was analyzed [43]. The analysis of the chemical composition material of the machined surface proves the existence of two defined zones: crater zone and crater-free zone. For the crater zone, the occurrence is noted of 4.12% of the oxygen element and 12.62% of the carbon element on the surface. The carbon element results from deposition of debris particles on the machined surface. The oxygen element stems from the rapid oxidation of the material with the coexistence of high temperature in the plasma channel and oxygen gas disassociated from the deionized water. For the crater-free zone, the oxygen and carbon elements are reduced considerably (from 4.12% to 2.31% and from 12.62% to 9.99%, respectively). These changes probably result from the dissolution of the material from the machined surface through electrochemical reaction. 

In the EDM process, it is worth underlining the significance of the temperature conditions occurring in the gap area. When using deionized water as working fluid, the bubbles are much smaller, while a single bubble is formed in the oil. Small bubbles move faster and do not significantly interfere with the erosion process [28,40]. In addition, the area proportion of the bubbles in the discharge gap is smaller in deionized water than in oil [28]. An analysis of the bubbles’ movement shows that an increase in the bubbles’ diameter is observed with an increase in the depth of the hole. This may lead to an increase in the bubble occupancy in the gap area and the weakening of the insulation strength of the dielectric liquid because, in general, the insulation strength of gas is much smaller than that of liquid [48]. The bubbles’ movements are also investigated in [29,49]. This analysis shows that at the beginning of consecutive pulse discharges, the bubbles rapidly remove the debris from the gap bottom. As the discharging continues, the bubbles’ ability to remove the debris weakens resulting in debris aggregation at the gap bottom and thus unstable machining [49]. In [29], the authors consider that the presence of bubbles increases the evacuation of eroded particles. If the kinematic viscosity of the dielectric fluid increases, the jump efficiency (defined as the ratio of the eroded particle number out the gaps at the end of the jump to the jump time) decreases, and a smaller number of particles is removed. However, the change of deionized water properties is not related to the value of dielectric fluid temperature, but in [21,29], the authors consider that the gaseous bubbles generated by the secondary discharges push the debris further along the axis of the electrode’s feed. Most of the debris is eventually driven out of the hole due to secondary discharges. In addition, the accumulated gas bubbles at the hole bottom can prevent electrochemical reaction [43].

The kind of working fluid also influences the process of forming the bubble around the plasma channel. As a result of a single impulse discharge, the molecules, atoms, ions and electrons formed as a result of evaporation, dissociation and ionization of the working liquid and the electrode material are compressed in a small bubble around the plasma channel [28]. The bubble diameter increases radially and peaks when the pressure inside the bubble reaches its minimum after the finished electrical discharge. Then, the bubble diameter is compressed to the initial diameter. In reality, the dielectric fluid viscosity results in the damping of the bubble diameter increase. It is noted that the bubble diameters in deionized water and oil are almost the same at the beginning of the diameter increase. After the damping, the bubble volume is significantly smaller in water than in oil. The reason factor is gas components of the bubble generated in oil, that is, gaseous hydrogen and hydrocarbon gases (such as methane, ethane, and acetylene), which are dissociated gases of hydrocarbon oil and cannot be recombined. The bubbles generated in deionized water are mainly composed of hydrogen and oxygen, which can be reversibly recombined into water. In addition, the damping coefficient in oil is higher than that in water, which is affected by the higher fluid viscosity in oil (2.4 × 10^−3^ Pa∙s) than in water (1.0 × 10^−3^ Pa∙s). In [50], authors also consider that the viscosity of the working fluid in the EDD process is relevant. The obtained results show that a low viscosity enables more effective flushing of the debris.

The above analysis of the papers shows a significant potential of the EDM process with the use of deionized water as working fluid, particularly for drilling high aspect ratio holes in difficult-to-cut materials. Using deionized water in the process improves the process performance (such as material removal rate, tool wear, surface roughness) and hole accuracy. In the EDD process, the influence of the deionized water properties on the process should be subjected to a more extensive analysis. Properties such as density, viscosity and electrical conductivity can have a major effect on the working fluid flow through the gap area and removal of erosion products. The change of the deionized water properties, especially the electrical conductivity, can contribute to remove an allowance in the similar range of ECM and EDM during the single pulse duration. The increase of the electrical conductivity can be influenced by the applied initial temperature of deionized water. The complexity of the phenomena occurring in the gap area during the process indicates the need for further experimental research.

This paper presents an analysis of the results of experimental research involving electrical discharge drilling (EDD) of aspect ratio holes in the Inconel 718 alloy with the use of deionized water as the dielectric fluid. The aim of the research was to check the impact of the properties of deionized water, such as electrical conductivity, and the working-fluid pressure on the process performance, dimensional and shape accuracy and inner surface integrity of the holes. The properties of deionized water were changed by applying an initial temperature of the deionized water.

The first part of the experimental research comprised an analysis of the impact of the process parameters (pulse time, current amplitude and discharge voltage) on process performance. The process performance was investigated in terms of linear tool wear, drilling speed and the accuracy of the holes (such as taper angle, aspect ratio of hole, side gap thickness). In order to examine the relationship between the process parameters and performance criteria, Analysis of Variance (ANOVA) techniques were applied. The process of drilling the holes was done on a sample consisting of two parts. The drilling was carried out at the junction of the sample parts. Once the parts of the sample were separated, an analysis of the dimensional and shape accuracy and quality of the inner hole surface was carried out. During the next part of the experiments, the impact of the initial temperature and the initial pressure of the deionized water onto the gap area on the conditions of fluid flow via the electrode channel on the volumetric flow rate and the Reynolds number was checked. The obtained through holes were subjected to the extensive analysis. Based on the previous series of experiments, the EDD of through holes for selected initial working fluid temperatures and pressure and selected optimum machining parameters was performed.

## 2. Materials and Methods

### 2.1. Materials

**Figure 1:**
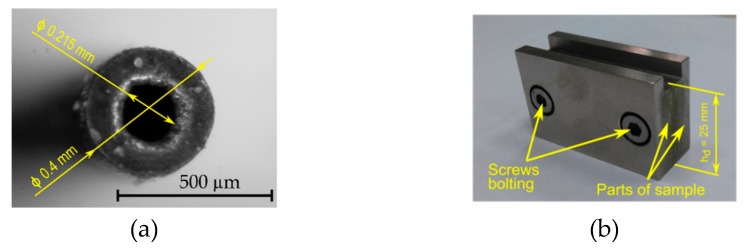
(**a**) Tool electrode tip and (**b**) photograph of the sample; *h_d_*—the maximum drilling depth.

The Inconel 718 alloy was used as the workpiece material and a tube-electrode was the tool (single-channel, made of copper) (Figure 1a). A special sample consisting of two parts was designed and produced as the workpiece for the purpose of the experimental test. The holes were drilled at the junction of the sample parts (Figure 1b). The chemical composition and the main physical-mechanical properties of the electrode material are presented in Table 1 and Table 2, respectively.

### 2.2. Experimental Procedure

**Figure 2:**
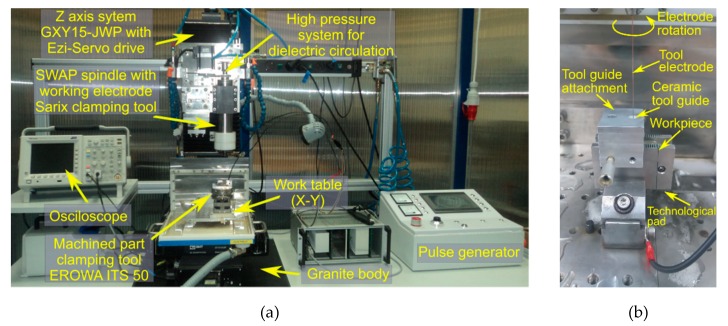
(**a**) Photograph of the test stand and (**b**) the experimental setup of the electrode guiding system.

First, electrical discharge drilling of deep holes was carried out on the experimental test stand shown in Figure 2a. In order to avoid problems with the drilling of through holes, an additional technological pad was applied on the underside of the sample (the thickness of the pad was 0.5 mm). In addition, to minimize the impact of electrode vibrations and the clamping eccentricity on the drilling process, an electrode guide system was applied (Figure 2b). The aim of the experimental research was to examine the influence of selected machining parameters on the dimensional accuracy of the hole, the machining efficiency and the tool electrode wear. Table 3 presents the data on the drilling process, and Table 4 shows the adopted ranges of values. The experiments were performed according to the theory of carrying out an experiment with the use of a three-level rotatable research plan that included 20 experimental tests, with six repetitions in the research plan center. The results of the experiments are shown in Table 5. The statistical techniques such as Analysis of Variance (ANOVA) were applied to investigate the relationship between the input and output parameters.

**Figure 3:**
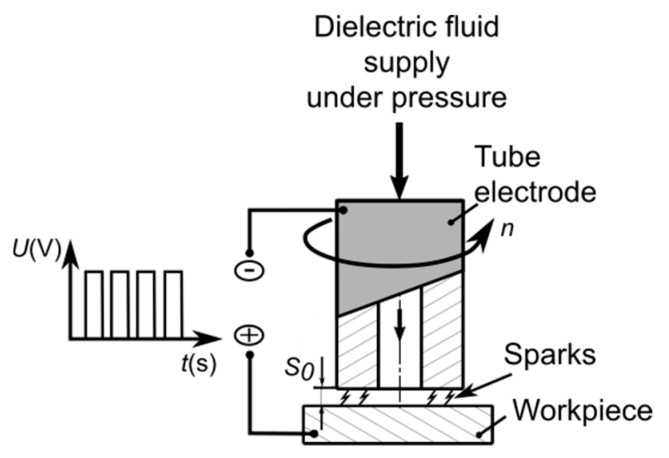
The scheme of the electrical discharge drilling (EDD) process.

The following constant parameters were assumed: initial interelectrode gap thickness (*S_0_* = 50 μm), inlet dielectric fluid pressure (*p* = 8 MPa), rotational speed of the clamp and the electrode (*n* = 400 rpm), drilling time of each hole (*t_drilling_* = 45 min), pulse off time (*t_off_* = *t_i_*) and deionized water as the dielectric fluid. Before each experiment was started, the temperature *T* (*T* = 297.15–316.15 °K) and the electrical conductivity *κ* of the deionized water were measured (*κ* = 3.8–6.8 µS/cm). The dielectric fluid was flushed down to the gap zone through the interior hole of the tube (Figure 3).

Measurements of the diameters along the hole’s depth were performed (five for each diameter) with the use of the K-401 stereo microscope with a Common Main Objective (CMO) Infinity optical system (Motic, Richmond, Canada) and equipped with a Moticam 2300 digital camera with MoticImages Plus system (Motic, Richmond, Canada). 

The difference between the relevant diameters of the two parts of sample was in the range 20–40 µm, and the calculated radius deviation of the tool electrode was 0.01 mm ((*D_tool guide_* − *D_tool_*)/2, *D_tool guide_*—inner diameter of the ceramic tool guide and *D_tool_*—outer diameter of the tool electrode). The above factors allowed the assumption of symmetrically drilled holes.

The drilling speed *v* is calculated from the following equation:

*v = h/t_drilling_*, (1)

where *h* is the hole depth, and *t_drilling_* is the drilling time.

The linear tool wear *TW* is calculated according to the formula:

*TW = (h_tool_/h)*·100%, (2)

where *h_tool_* is the shortening of the electrode.

The aspect ratio hole *AR* is given by the following equation:

*AR = h/D_average_*, (3)

where *D_average_* = (*D_top_* + *D_bottom_*)/2 is the average of the hole diameters, *D_top_* is the average top diameter, and *D_bottom_* is the average bottom diameter (values of *D_top_* and *D_bottom_* are the mean values from five measurements). 

The taper angle *tap_α_* is calculated from the following equation:

*tap_α_ = (D_top_ – D_bottom_)*/2*h*. (4)

The side gap thickness *S_b_* is calculated according to the formula:

*S_b_ = (D_top_ – D_tool_)*/2. (5)

### 2.3. Experimental Procedure for the Flow of the Deionized Water through the Electrode Channel

**Figure 4:**
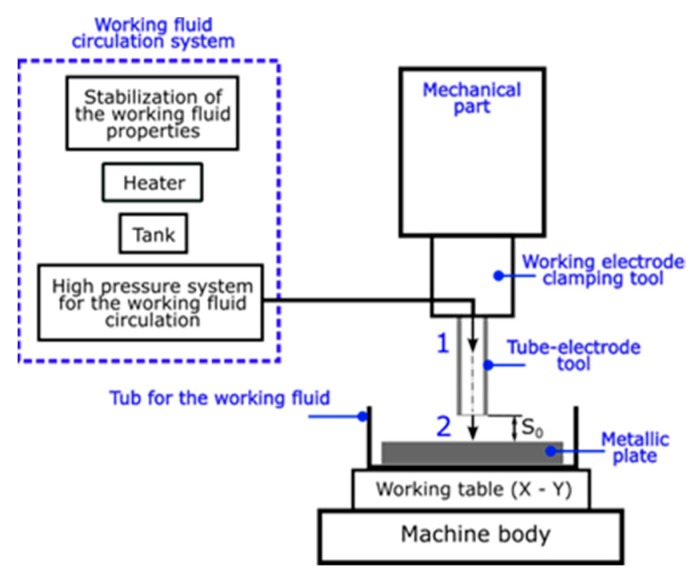
Test stand diagram.

The second part of experimental research included an analysis of the working fluid’s flow through the electrode channel. Deionized water was applied as the working fluid. The aim of the experiment was to examine the impact of the initial working-fluid temperature *T* the and initial working-fluid pressure *p* on the volumetric flow rate *Q* and the Reynolds number *Re*. Table 6 presents the thermal properties of the working fluid according to its temperature. In Table 3, the boiling point of the working fluid (*T* = 373.15 °K) is also specified. It is related to reaching the critical conditions in the gap zone (then the deionized water temperature reaches the boiling point), which ensures the occurrence of electrical discharges in a single impulse time. The tool electrode, which was applied, had the same shape and the same dimensional parameters as in the EDD experiment (cross-sectional area of the electrode’s channel *S* = 0.0363 mm^2^, length 150 mm). The gap thickness *S_0_* was 100 µm. During the experiment, the working fluid was flushed down the interior hole of the tube with the pre-set initial pressure and pre-set initial temperature (designation 1, Figure 4). Next, the working fluid flowed onto the flat surface of a metallic plate (designation 2, Figure 4). 

The research was performed according to the experiment theory, with the use of a two-level rotatable research plan that included 11 experimental tests with three repetitions in the research plan’s center. Table 7 presents the adopted parameter ranges. The results of the experiment are shown in Table 8.

In a cylindrical tube, the laminar flow is considered with the Reynolds number of below 2300. For the values of the Reynolds number above 2300, the turbulent flow is estimated.

The volumetric flow rate *Q* was determined experimentally. In order to calculate the flow conditions, the Reynolds number *Re* was used, which is defined in Equation (6)

*Re = (v_f_∙r)/ γ*, (6)

where *r* was the radius of the channel diameter in the tool electrode, *γ* was the kinematic viscosity of the working fluid (value applied for the relevant working fluid temperature according to Table 6), and the mean flow rate velocity of the fluid *v_f_* was calculated from the following formula:

*v_f_ = Q/S*, (7)

where *S* was cross-sectional area of the electrode’s channel.

In order to estimate the impact of the initial working-fluid temperature *T* and the initial working-fluid pressure *p* on the volumetric flow rate *Q* and the Reynolds number *Re*, the *Matlab* software was employed. A second-degree polynomial (with constant, linear, interaction and square terms) was used as the function. The regression equations obtained for the relationships *Q* (*T*, *p*) and *Re* (*T*, *p*), are described as the following equations:

*Q* (*T*, *p*) = −3.272∙10^−5^ + 2.175∙10^−7^∙*T* + 1.349∙10^−7^∙*p* − 5.867∙10^−10^∙*T∙p* − 3.561∙10^−10^∙*T*^2^ + 7.574∙10^−9^∙*p*^2^, (8)

*Re* (*T*, *p*) = −55753.573 + 359.136∙*T* − 921.200∙*p* + 2.681∙*T∙p* − 0.560∙*T*^2^ + 24.147∙*p*^2^. (9)

The values of the coefficient *R*^2^ (*R*—square Statistic) and *R^2^* adjusted (Adjusted *R*—square Statistic) are high (Table 9), which can confirm that the fitted quadratic models are statistically significant for the analyzed relationships of *Q* (*T*, *p*) and *Re* (*T*, *p*). The values of “*p*-Value” are less than 0.05 (i.e., 95% of the confidence level), which confirms that the models obtained are statistically significant.

## 3. Results

### 3.1. Results of the EDD Analysis

In order to determine the impact of the process parameters on the process performance, the ANOVA analysis was employed. The regression equations *v* (*U, t_i_*, *I*), *TW* (*U, t_i_*, *I*), *tap_α_* (*U, t_i_*, *I*), *AR* (*U, t_i_*, *I*) and *S_b_* (*U, t_i_*, *I*) were determined by the relevant Equations (10)–(14):

*v* (*U, t_i_*, *I*) = 19.588 + 0.1033∙*U* + 0.0073∙*t_i_* − 13.5625∙*I* + 2.1517∙*I^2^* − 0.0001∙*U*∙*t_i_*, (10)

*TW* (*U, t_i_*, *I*) = 26.0443 + 0.4937∙*U* − 0.0606∙*t_i_* − 10.0885∙*I* − 0.0012∙*U*∙*t_i_* + 0.0529∙*t_i_*∙*I*, (11)

*tap_α_* (*U, t_i_*, *I*) = −0.015524 − 0.00003∙*U* + 0.00005∙*t_i_* − 1∙10^−7^· *t_i_^2^* + 0.00155∙*I* − 8∙10^−6^∙*t_i_*∙*I*, (12)

*AR* (*U, t_i_*, *I*) = −39.3292 + 1.1504∙*U* − 0.0055∙*U^2^* + 0.0152∙*t_i_* − 0.000001∙*t_i_^2^* + 1.561∙*I*, (13)

*S_b_* (*U, t_i_*, *I*) = 375.2799 − 8.2966∙*U* + 0.0422∙*U^2^* + 0.2552∙*t_i_* + 34.0705∙*I* − 0.0599∙*t_i_*∙*I*. (14)

The Equations (10)–(14) involve the drilling parameters with the regression coefficients at the significant level of “*p*-Value” *<* 0.05. 

The shape of the holes obtained is characterized by reverse conicity. The value of the top diameter is lower than that of the bottom diameter. This may result from process instability due to the accumulated debris at the hole bottom and an insufficient dielectric flow through the machining gap area.

**Figure 5:**
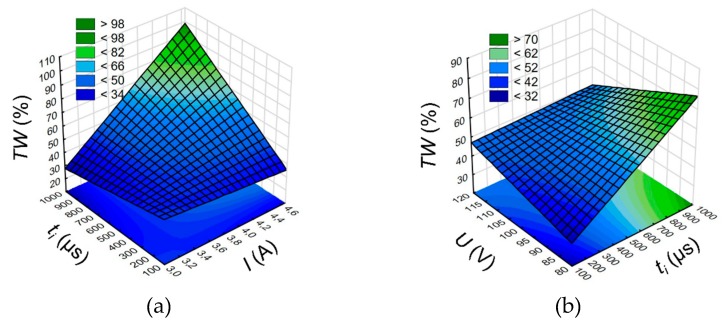
(**a**) Relationship between the linear tool wear *TW* and the pulse time *t_i_* and current amplitude *I*, *U* = 100 V and (**b**) discharge voltage amplitude *U*, *I* = 3.83 A.

**Figure 6:**
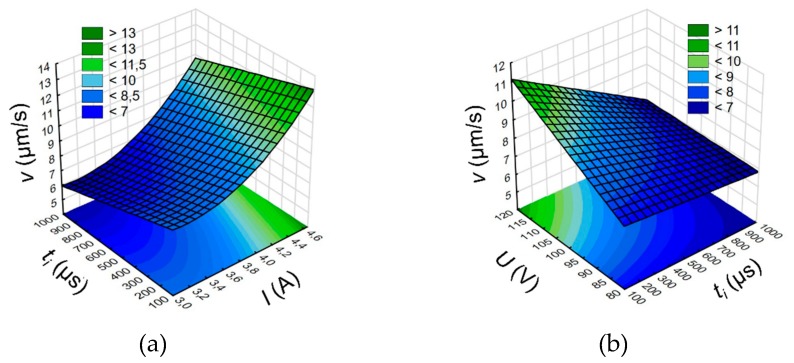
Relationship between the drilling speed *v* and the pulse time *t_i_* and (**a**) current amplitude *I*, *U* = 100 V and (**b**) discharge voltage amplitude *U*, *I* = 3.83 A.

The analysis of the results shows that the high linear tool wear (*TW* > 60%) takes place where a higher current amplitude (*I* > 4.32 A) and a higher discharge voltage (*U* > 100 V) are applied. As a result, the amount of the removed material depended mainly on the energy in a single discharge. Higher *U* and *I* cause a higher single discharge energy, and the removal of a higher amount of material from both electrodes. The insufficient working fluid flow can then cause excessive accumulation of debris at the hole bottom. A lower interelectrode gap can contribute to secondary discharges between the accumulated debris and the electrode’s front, causing excessive tool wear. Additionally, a higher *TW* (above 80%) is observed for longer pulse time *t_i_* = 818–999 µs and a higher current amplitude *I* = 4.32 – 4.65 A (Figure 5a). A longer pulse time can result in a longer time of occurrence of electrical discharges in a single pulse. The occurrence of secondary discharges (between debris and hole sidewall) can also be longer, which results in additional wear of the tool electrode. An increase in the pulse time has an irrelevant effect on the drilling speed *v* (Figure 6a). However, an increase in the discharge voltage *U* and a decrease in the pulse time *t_i_* cause a decrease in the linear tool wear (Figure 5b) and an increase in the drilling speed (Figure 6b). In the case of application of *U* = 100–120 V and *t_i_* = 100–282 µs, the electrochemical dissolution in a single impulse can be enhanced, which improves the drilling speed. A lower value of the pulse time can reduce the time of occurrence of electrical and abnormal discharges, thus decreasing the linear tool wear. Additionally, based on the ANOVA analysis for *TW,* the selected relevant parameter is the discharge voltage *U* (“*p*-Value” = 0.171). The results of the ANOVA analysis for drilling speed *v* and linear tool wear *TW* are given in Supplementary section S1.

**Figure 7:**
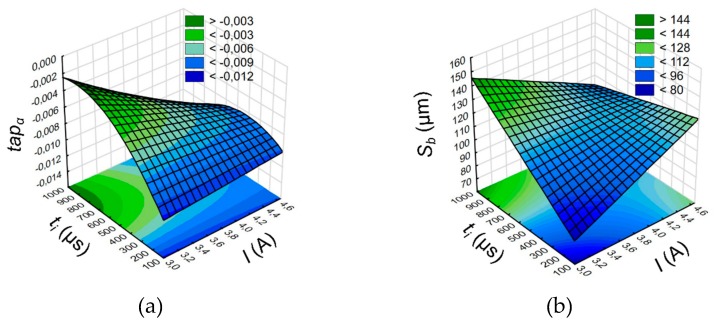
(**a**) Relationship between the taper angle *tap_α_*, current amplitude *I* and pulse time *t_i_*; (**b**) relationship between the side gap thickness *S_b_*, current amplitude *I* and pulse time *t_i_*; *U* = 100 V.

The “-” sign for taper angle *tap_α_* indicates a reverse conicity of the hole. For a longer pulse time *t_i_* = 818–999 µs and a lower current amplitude *I* = 3–3.33 A, a drop in *tap_α_* was noted (Figure 7a). It is related to the increase in the side gap thickness *S_b_* (Figure 7b). A longer pulse time can cause a longer time of occurrence of electrical and abnormal discharges in a single pulse, which extend the bottom diameter of the hole. An increase in the side gap causes a smaller difference between the top and bottom diameters which decreases *tap_α_*. An increase in *S_b_*, on the other hand, is observed for a lower pulse time *t_i_* = 100–282 µs, which was applied and an increase in the current amplitude *I* (Figure 7b). This may be due to excessive electrochemical dissolution in a single pulse. Electrochemical dissolution also takes place during the flow of the working fluid outside of the hole. An increase in the side gap thickness is also noticed for the higher discharge voltage *U* = 100–120 V, which was applied. The higher discharge voltage and the higher electrical conductivity of deionized water (*κ* = 3.8–6.8 µS/cm) could significantly increase the electrochemical dissolution in a single impulse. Based on the ANOVA analysis for the *tap_α_*, the discharge voltage is regarded as significant parameter (“*p*-Value” = 0.129), but for the *S_b_*, the discharge voltage (“*p*-Value” = 0.233) and the current amplitude (“*p*-Value” = 0.717). The results of the ANOVA analysis for the taper angle *tap_α_* and the side gap thickness *S_b_* are given in Supplementary section S1.

**Figure 8:**
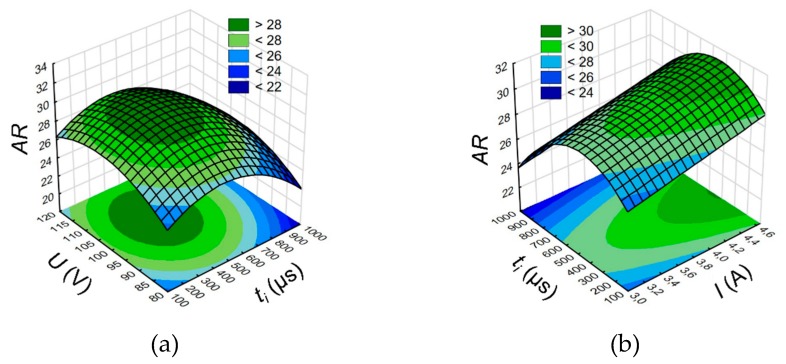
(**a**) Relationship between the aspect ratio *AR* and the pulse time *t_i_* and (**a**) discharge voltage amplitude *U*, *I* = 3.83 A and (**b**) current amplitude *I*, *U* = 100 V.

The aspect ratio hole *AR* is related to the drilling speed *v,* which results from the application of the same drilling time for each test. An analysis of the results shows that the extending of the pulse time (Figure 8a,b) causes a decrease in *AR*. For a longer pulse time in a single impulse, a longer time of electrical and secondary discharges may also occur. As a result, a considerable amount of debris is accumulated at the hole bottom. Where the working fluid flow is insufficient, the process is unstable which reduces the material removal rate. The optimum pulse time affecting the *AR* increase is within the range of 500–818 µs (*AR* > 26). The ANOVA analysis shows that the process parameters such as the discharge voltage *U* (“*p*-Value” = 0.116), the pulse time *t_i_* (“*p*-Value” = 0.09) and the current amplitude *I* (“*p*-Value” = 0.11) should be also regarded as a significant parameter. The results of the ANOVA analysis for the aspect ratio hole *AR* is given in Supplementary section S1.

### 3.2. Results Analysis of the Working Fluid Flow in the Electrode Channel

When deionized water is used as dielectric to obtain electrical discharges in a single pulse, the critical conditions must be achieved within the gap area involving a significant number of gas bubbles. For gas bubbles to appear within the gap area, the deionized water must reach the boiling point (373.15 °K). An increase in the temperature of the deionized water also changes its physical properties such as electrical conductivity, density or viscosity. Density and viscosity values decrease as the temperature increases, while electrical conductivity increases as the temperature increases (Table 6). Lower density and viscosity should improve the working fluid flow through the gap area. However, the higher electrical conductivity of deionized water should reinforce the electrochemical reactions in a single pulse. For this reason, the initial working-fluid temperature is an important parameter influencing the flow conditions and the thermal conditions within the gap area. The other important parameter influencing the fluid flow is the initial working-fluid pressure. 

**Figure 9:**
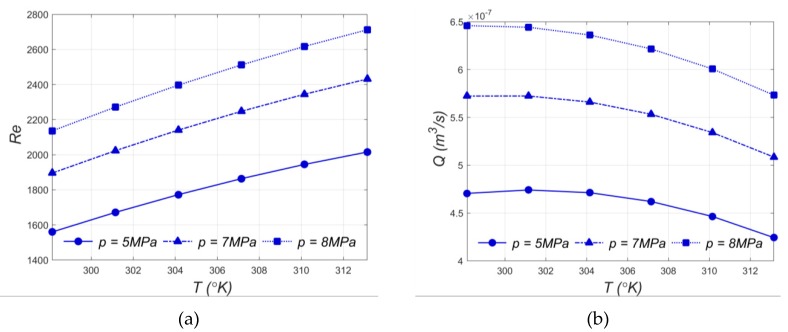
Impact of the initial temperature of fluid *T* and initial working fluid inlet pressure *p* on (**a**) the Reynolds number *Re* and (**b**) the volumetric flow rate *Q*.

The analysis of the flow results for an electrode with an outer diameter of 0.4 mm shows the Reynolds number below 2300 for *p* = 5 MPa and *p* = 7 MPa, which were applied (Figure 9a). Then, the fluid flow assumes the laminar flow conditions. In the case where the initial working-fluid pressure applied is *p* = 8 MPa and the initial working-fluid temperature is above 300 °K, the Reynolds number is above 2300 and a turbulent flow should be considered. The analysis of the results indicates that the volumetric flow rate is about 1.4 times higher in the case where *p* = 8 MPa is applied than for *p* = 5 MPa (Figure 9b). This may be related to the viscosity and density of deionized water, whose values decrease with temperature increases. Lower viscosity and lower density of deionized water ensure a faster fluid flow through electrode channel. 

The consideration of turbulent flow conditions for *p* = 8 MPa and *T* > 300 °K is related to a higher flow rate velocity, which enhances the evacuation of the eroded material from the gap area. It is worth underlining that a turbulent flow may be accompanied by whirls, which may prevent correct working fluid flow with eroded particles and reduce process stability.

### 3.3. Results of the Analysis of the Through Holes Obtained for the Experiment 1

**Figure 10:**
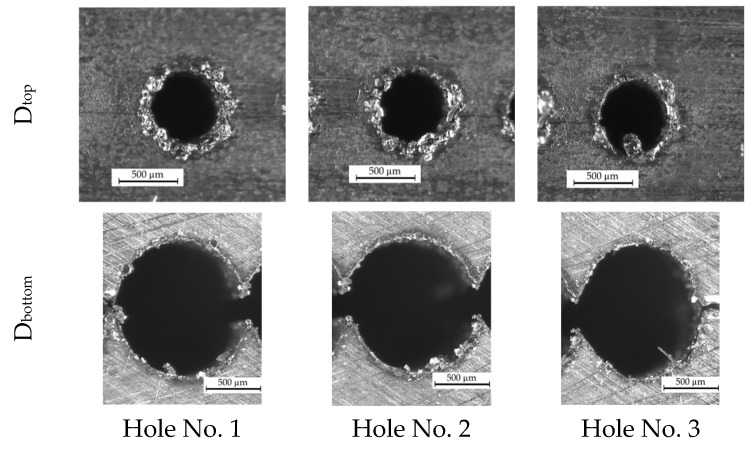
Through holes drilled with the application of the same machining parameters: *t_i_* = 282 µs, *U* = 120 V, *I* = 4.32 A.

The experimental research enabled the obtaining of through holes. The sixth test (machining parameters such as *t_i_* = 282 µs, *U* = 120 V, *I* = 4.32 A) and the eighth test (machining parameters such as *t_i_* = 818 µs, *U* = 120 V, *I* = 4.32 A) provided the drilling of the through holes. In the case of the eighth test, the linear tool wear was significant (*TW* > 70%), which is due to the application of a longer pulse time. The time of electrical (and secondary) discharges in a single impulse could be too long which resulted in the increased electrode tool wear. Therefore, the machining parameters of the sixth test were chosen to drill additional through holes (two additional through holes were made) (Figure 10). It is worth to underline that the electrical conductivity and the temperature of deionized water while drilling the three through holes were in the range *κ* = 5.7–6.3 µS/cm and *T* = 302.15–308.15 °K, respectively. These values of *T* and *κ* were higher than for the remained tests. 

**Figure 11:**
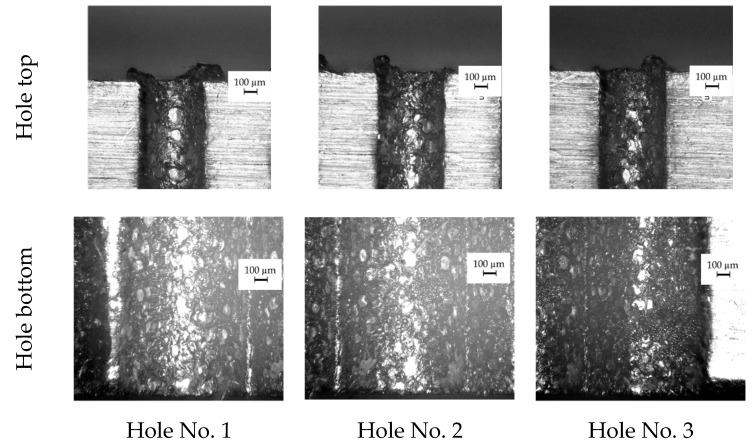
Images of the longitudinal section of three through holes at the hole top and bottom.

**Figure 12:**
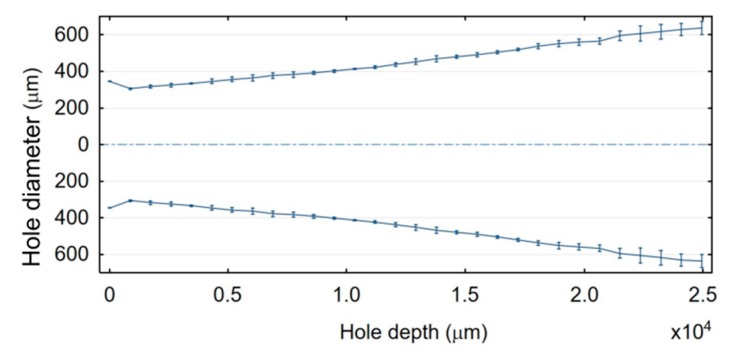
Profile of the through hole along its depth.

The dimensional accuracy and the inner surface homogeneity of the drilled holes are insufficient (Figure 10 and Figure 11). The bottom diameter is greater on average by 43% than the top diameter. The profile of the hole based on the average measurements of the diameters along the hole depth for three through holes is presented in Figure 12. The diameter increases along the hole length (from the hole’s top to its bottom). Each diameter is larger than the previous one by about 3% on average. This results from an insufficient working fluid flow through the gap area and insufficient debris removal. Secondary discharges can occur between the accumulated debris and the hole sidewall. When the hole depth is significant (above 10,000 µm), the phenomenon of secondary discharges may be excessive. When the hole depth is near 25,000 µm, the bottom diameter is almost twice as large as the top diameter. This confirms that the eroded material is not fully removed and accumulates at the hole bottom.

**Figure 13:**
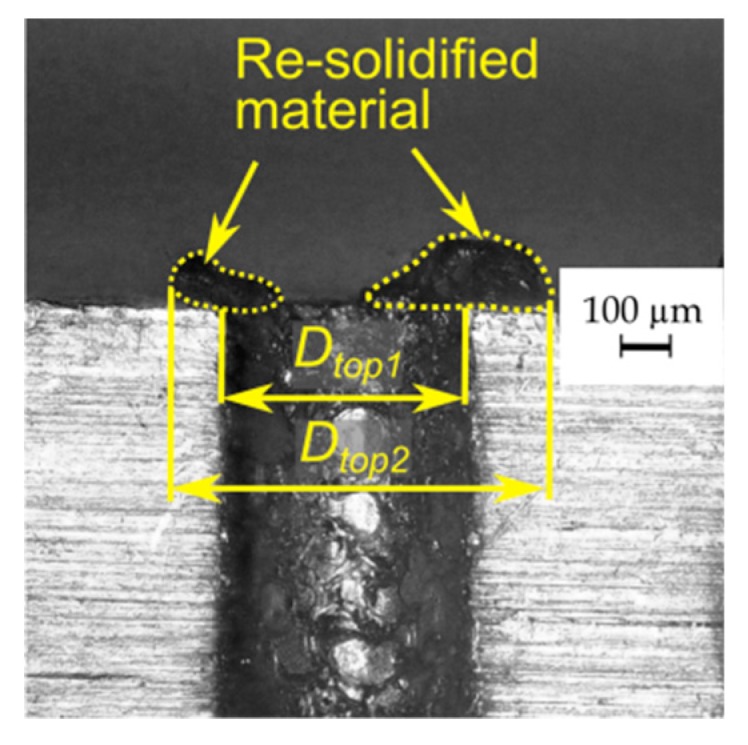
Image of the through hole top of one part of the sample after separation.

On the edge of the bottom diameter, burrs occur. But on the surface of the top diameter, there is a significant amount of re-solidified material and the heat affected zone (Figure 10). After separating the parts of the sample, two top diameters of the hole *D_top1_* and *D_top2_* (where *D_top2_* > *D_top1_*) can be observed (Figure 13). The determination of the *D_top2_* diameter follows from the unremoved and re-solidified material deposited on the hole edge. The average diameter of re-solidified material is larger by about 180 µm than *D_top1_*. The top diameter, without the re-solidified material (*D_top1_*), should constitute the correct top diameter. The occurrence of the re-solidified material may result from an appropriate working fluid flow at the beginning of the process. The fresh dielectric with a lower temperature could affect the eroded material solidifying it too fast on the surface before it was removed.

**Figure 14:**
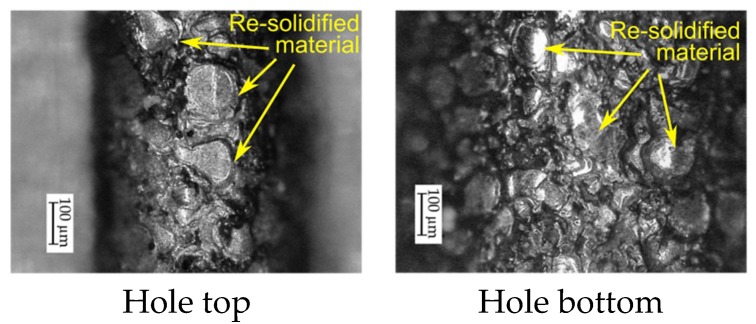
Images of the internal surface of the through hole.

At the beginning of drilling the hole, the working fluid flow should be correct. However, on the inner surface of the hole top, a significant amount of melted and re-solidified material is observed (Figure 14), which indicates an insufficient working fluid flow in the gap area. In order to understand this phenomenon, it may be helpful to focus on the conditions contributing to the occurrence of electrical discharges in a single impulse when deionized water is used as a dielectric. The factors which influence these conditions are mainly the discharge voltage and the impulse duration. 

**Figure 15:**
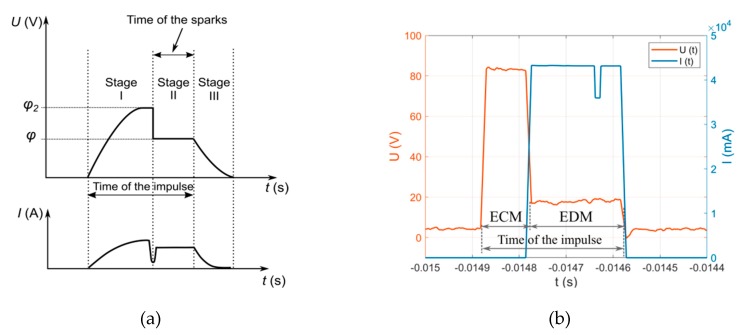
(**a**) Typical voltage and current waveform in a single impulse during the Electrochemical Discharge Machining (ECDM) process; (**b**) the recorded voltage and current waveform for the through hole parameters.

At the beginning of the pulse time (Stage I, Figure 15a), the voltage between the electrodes and the current amplitude increases gradually. The allowance is then removed by electrochemical dissolution. The application of a higher voltage and a working fluid with higher electrical conductivity can contribute to reinforcing the electrochemical dissolution. When the voltage increases (up to *φ_2_*), hydrogen is generated on the tool electrode, which leads to the formation of a gas film around the tool. The bubbles increase their diameters over time until the critical size is achieved. When the number and size of the hydrogen bubbles are sufficient, the resistance on the tool electrode–workpiece interface increases substantially due to the constriction effect. This contributes to increased ohmic heating of the working fluid within the area, causing the bubbles to evaporate. At the critical condition, a large number of gas bubbles cover the maximum possible active surface area of the tool electrode, leading to its blanketing (i.e., isolation between the electrode surface and the working fluid). Consequently, the current decreases in a very short time [55]. At the beginning of Stage II, a sudden decrease in voltage occurs (up to *φ*) and, at the same time, the current amplitude increases, which may indicate the occurrence of discharges and the allowance is removed in a manner typical of the EDM process. At the end of the pulse time, due to the electric capacity effect, the voltage between the electrodes gradually decreases to zero (Stage III, Figure 15a) [56,57]. The voltage applied should be high enough, and the pulse time should be long enough for the discharges proper to occur in a single impulse. The recorded voltage of the discharge and current amplitude in a single impulse characterizes the typical waveform for the ECDM process (Figure 15b). There is no current increase at the beginning of the pulse time due to the application of the pulse generator setting up the EDM process.

**Figure 16:**
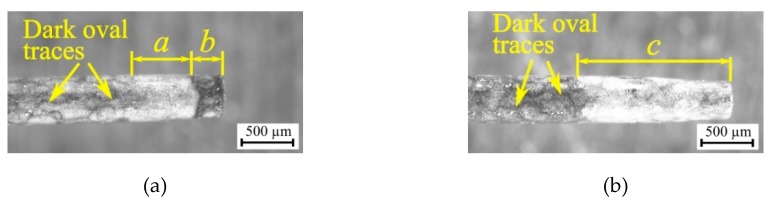
Photographs of tool electrode wear after drilling the holes with the machining parameters applied: (**a**) *t_i_* = 282 µs, *U* = 120 V, *I* = 4.32 A; (**b**) *t_i_* = 550 µs, *U* = 100 V, *I* = 3.83 A.

The observation of the surface on the hole top and near the hole bottom can confirm the occurrence of a considerable number of gas bubbles within the gap area, which were not fully removed. However, many more small bubbles might have occurred at the bottom of the hole because of incorrect fluid flow. For this reason, oval traces of re-solidified material on the surface of the hole bottom are smaller than on the surface of the hole top. The oval shape of the traces may be due to the places where the electrical discharges occurred. According to [58], electrical discharges can occur on small areas between bubbles (bubble bridges) and the workpiece surface. This may also explain the occurrence of dark oval traces on the surface of the electrode tool (Figure 16). The gas bubbles creating the gas film on the electrode come and go all the time. For this reason, only darker traces of electrical discharges remained on the electrode surface, and the electrode material did not erode. The lower thermal conductivity of Inconel 718 could also cause a significant amount of heat to remain in the processing area in case of abnormal flow of the liquid. As a result, a considerable number of small bubbles could be present all the time within the gap area creating favorable conditions for the occurrence of electrical and secondary discharges. 

The shape of the tool electrode after the drilling did not change significantly. The tool electrode tip wore to a lesser degree after the drilling of the through hole (machining parameters: *t_i_* = 282 µs, *U* = 120 V, *I* = 4.32 A, electrode tip diameter smaller by about 10%) than after the drilling with the machining parameters in the research plan center (*t_i_* = 550 µs, *U* = 100 V, *I* = 3.83 A, electrode tip diameter smaller by about 20%). The length of the electrode tip surface, which was worn, was also larger after the drilling with the parameters in the research plan center (*c* = 1229 µm, *t_drilling_* = 2700 s, *h* = 22,044 µm) than after the drilling of the through hole (*a + b* = 916 µm, *t_drilling_* = 2316 s, *h* = 25,000 µm) (Figure 16a,b). The dark fragment on the electrode tip (*b* = 269 µm, Figure 16a) may be the result of a significant amount of the eroded particles at the hole bottom and the occurrence of the critical conditions including the generation of gas bubbles and the secondary discharges within the gap area. In addition, the presence of high temperature at the gap area and application of deionized water effected the formation of copper oxide, which provoked a grey–blue color on the electrode surface after the drilling process. 

### 3.4. Results of the Analysis of the Through Holes Obtained for the Experiment 2

**Figure 17:**
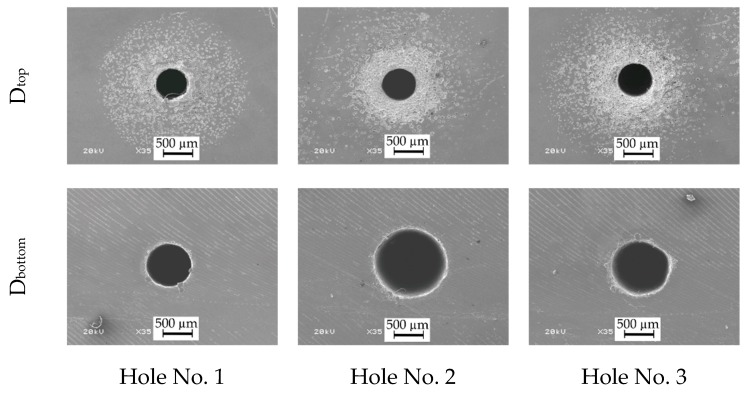
Through holes drilled in the Inconel 718 alloy.

Based on an analysis of the results of electrical discharge drilling and the working fluid flow through the electrode channel, the experimental research into the EDD of through holes in the Inconel 718 alloy was done. In contrast to the previous experiment, the following was applied: longer pulse time (*t_i_* = 500 μs), the given initial working-fluid temperature (*T* = ~313.15 °K) and higher electrical conductivity of deionized water (*κ* = 7.2–12.8 µS/cm). Three tests were performed with the same machining parameters (Figure 17).

The significant electrical conductivity of deionized water caused the corrosion of the workpiece surface. Serious damage is observed on the surface around the top diameter (Figure 17). The thickness of the damaged surface is about 550 µm. 

**Figure 18:**
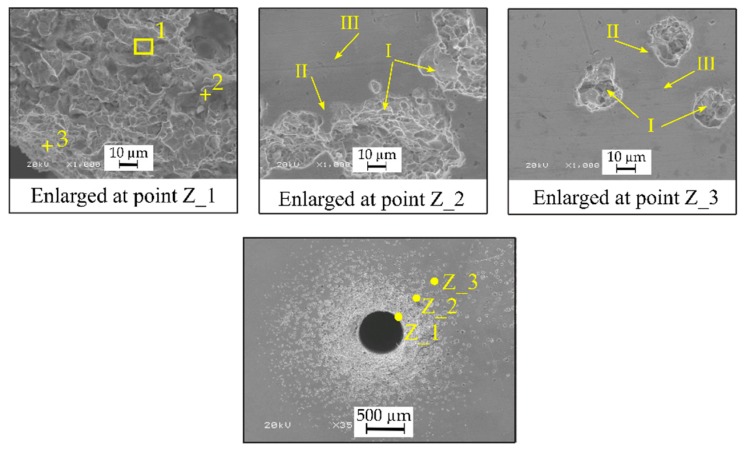
SEM images surface around the *D_top_* of hole No. 3.

The damaged surface around the hole was subjected to a qualitative analysis with the use of a scanning electron microscope (SEM) with an energy dispersive spectroscopy system (EDS), manufactured by JEOL Ltd. (Tokyo, Japan). The analysis was made in three marked zones: zone 1 (Z_1), zone 2 (Z_2) and zone 3 (Z_3) (Figure 18). A surface analysis (for deep areas, designation 1 in Figure 18, enlarged at point Z_1) and a spot analysis (for the flat areas, designation 2 and 3 in Figure 18, enlarged at point Z_1) were performed.

**Figure 19:**
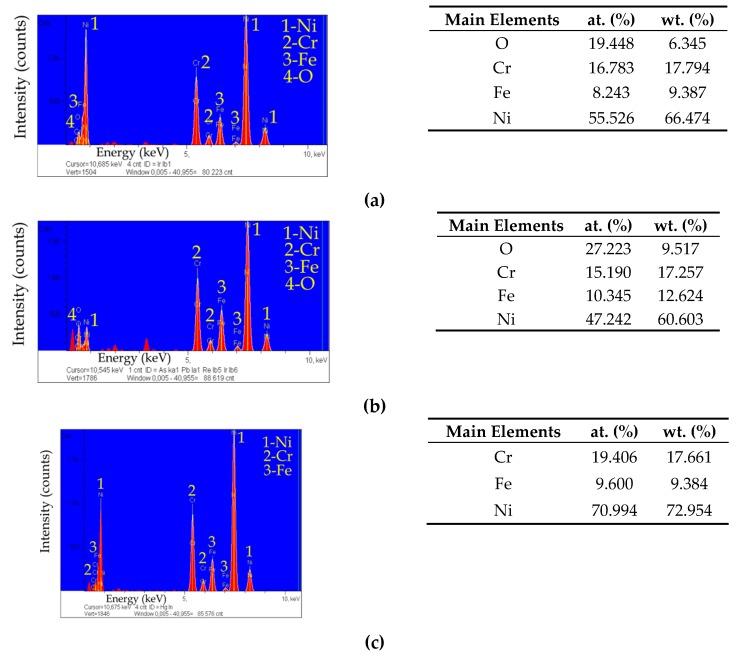
Chemical composition of the surface around the top diameter as determined by EDS: (**a**) zone of cavities, (**b**) darker zones near the cavities, (**c**) brighter zones between the cavities.

On the damaged surface, zones cavities (Figure 18, enlarged at points Z_2 and Z_3, designation I), darker zones near the cavities (Figure 18, enlarged at points Z_2 and Z_3, designation II) and brighter zones between the cavities (Figure 18, enlarged at points Z_2 and Z_3, designation III) can be observed. The analysis carried out for the three areas (marked as Z_1, Z_2, Z_3) indicates the occurrence of the average percentage volume of the main components of the Inconel 718 alloy: Ni-67.47 wt.%, Cr-17.66 wt.%, Fe-9.45 wt.% (Figure 19a–c). Additionally, in the cavities (Figure 19a) and darker zones (Figure 19b) the presence of oxygen was detected. This results from the use of deionized water. In high temperature conditions, a passive layer might have formed on the workpiece surface due to oxidation reaction. The passive layer might have prevented excessive electrochemical dissolution when deionized water was flowing out of the hole. 

Observation of the damage that surfaced indicates that electrochemical reactions were the most excessive while the fluid was flowing from the electrode channel (the initial water temperature was about 313.15 °K). The splashing working fluid further on the workpiece surface lost its heat, and its electrical conductivity decreased. This can be inferred from the size of the cavities near and farther away from the hole edge. The cavities located farther are less numerous and smaller (Figure 18, enlarged at points Z_2 and Z_3).

The EDS analysis of the surface near the hole edge indicates the absence of oxygen (Figure 18, enlarged at point Z_1). On this area, the material was removed by simultaneous interaction of electrochemical dissolution and electrical discharges in a single pulse.

**Figure 20:**
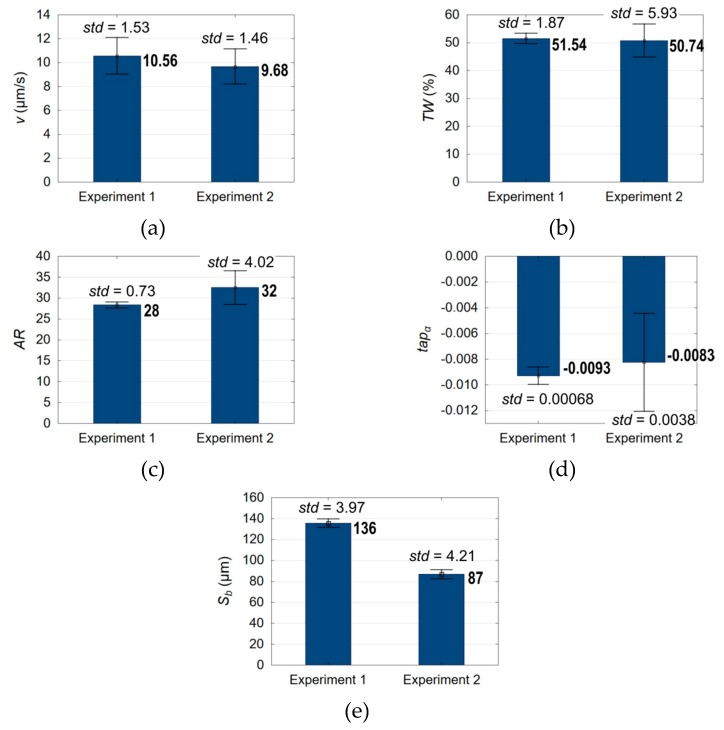
Comparison of the process performance in Experiments 1 and 2 for: (**a**) the drilling speed *v*, (**b**) the linear tool wear *TW*, (**c**) the aspect ratio hole *AR*, (**d**) the taper angle *tap_α_*, and (**e**) the side gap thickness *S_b_*; *std* – the value of the standard deviation.

Based on Equations (1)–(5), the process performance comprising the drilling speed *v*, the linear tool wear *TW*, the aspect ratio hole *AR*, the taper angle *tap_α_* and the side gap thickness *S_b_* were determined (in Figure 20a–e as Experiment 2). The analysis of the results showed that the drilling speed, the linear tool wear and the taper angle are at a similar level in comparison to the previous experiment (Figure 20a,b,d, respectively). The aspect ratio hole and the side gap thickness improved by about 15% and 40%, respectively (Figure 20c,e).

**Figure 21:**
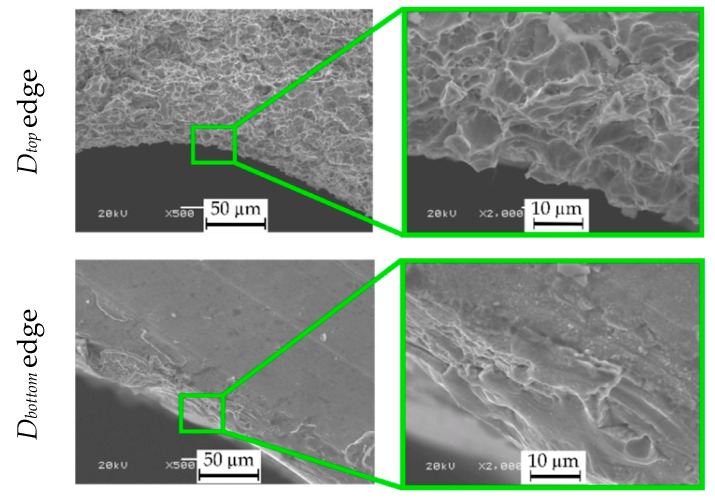
SEM images of the surface of *D_top_* and *D_bottom_* edge, hole No. 3.

The increase in *AR* results from obtaining a lower average top diameter value. The edge of the top diameter is free of re-solidified material, a heat-affected-zone and burrs, which improves its accuracy (Figure 17 and Figure 21). This may result from excessive electrochemical dissolution in a single pulse due to a higher electrical conductivity of deionized water resulting from the application of a higher initial working-fluid temperature (about 313.15 °K). In addition, the higher discharge voltage (*U* = 120 V) additionally enhances the electrochemical reactions in a single pulse. The longer pulse off time (about twice as long as during Experiment 1), on the other hand, results in more efficient removal of the eroded material. The conditions in the working gap might also have improved due to a higher fluid flow because of lower viscosity and lower density of deionized water. A smaller top diameter influences the improvement of *AR* and *S_b_*.

The surface around the top diameter is smoother and without microcracks. The bottom diameter is characterized by a significantly smaller number of burrs than after Experiment 1 (Figure 17 and Figure 21). The white layer around the bottom diameter is present, but the diameter quality is enhanced. The average value of the bottom diameter is still large (about 1000 µm) in comparison with the average value of the top hole diameter (about 600 µm), what causes the hole conicity (Figure 20d). The increased bottom diameter result from the accumulation of debris at the hole bottom and the occurrence of abnormal discharges. Additionally, the application of a higher initial working-fluid temperature (about 313.15 °K) and a higher initial working-fluid pressure (*p* = 8 MPa) could cause a turbulent flow and the occurrence of whirls at the hole bottom. The presence of whirls could cause difficulties removing debris and bubbles from the bottom of the hole. 

In [43], it was observed that gas bubbles, which were occurring, can prevent electrochemical dissolution. The higher electrical conductivity of deionized water and higher discharge voltage have contributed to achieving critical conditions faster in the gap area and faster occurrence of the bubbles. The higher electrical conductivity also has caused the increase in the fluid temperature by generating Joule heat. The considerable number of gas bubbles with the gap area may explain the absence of drilling speed improvement. However, in [21,29], it is considered that the bubbles push the debris and help remove them from the gap. However, the extending diameter along the hole depth attests to the occurrence of a significant amount of debris within the side gap area near the hole bottom. This may confirm the occurrence of whirls resulting from a turbulent flow. The whirls could hinder proper working fluid flow and, additionally, push the unremoved debris towards the corner of the hole bottom.

**Figure 22:**
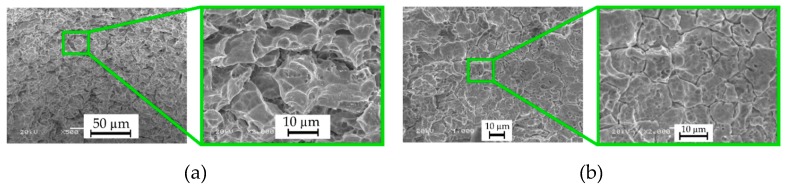
SEM images of (**a**) the inner surface of the hole (hole No. 3) and (**b**) the surface after electrochemical dissolution with the use of deionized water (machining parameters: *U* = 25 V, *t_i_* = 300 μs).

The inner hole surface is significantly less rough and more homogenous (Figure 22a), in comparison with the surface after the electrochemical machining process only (Figure 22b) and after the previous experiment (Figure 10). There are no micro-craters, re-solidified material or micro-cracks on the surface. The surface structure is similar to the structure that was obtained around the hole top. This is due to parallel removal of the material as a result of electrochemical reactions and electrical discharges. When the working fluid was flowing out of the hole, electrochemical dissolution might have occurred as well, which additionally improved the homogeneity of the inner surface of the hole.

## 4. Discussion

The results analysis of the experimental research indicates that the applied higher temperature of deionized water (*T* = ~313.15 °K), used as working fluid, effected the improvement of shape dimensional drilling the through holes. The inner surface homogeneity of holes also significantly improved. On the inner of the through holes observed a lack presence of re-solidified material, of which a significant amount was present on the surface after the first part of the research (Figure 10 and Figure 13). This is result using higher temperature of deionized water had an influence in increasing electrical conductivity during the experiment 2. The electrical conductivity increase caused the enhanced electrochemical dissolution at the beginning of the single pulse time (removing an allowance in the similar ranges by ECM and EDM during the single pulse duration). The reinforced electrochemical dissolution was enough to remove the erosion craters and to avoid extending the top diameter of holes simultaneously. In addition, using of the longer pulse off time (*t_off_* = 500 µs, above twofold longer in comparison to the first experiment), could also cause to avoid re-solidified material on the hole edge. During the experiment 1 the pulse off time (*t_off_ = t_i_*) equaled 282 µs, which was too short time a fresh working fluid to flow onto the gap area. The insufficient decrease in the temperature of the working fluid in the gap area could cause a too fast reoccurrence of conditions enough to spark (particularly, in case of using the higher initial working–fluid temperature *T* = 313.15 °K). As the result, the amount of re-solidified material could be occurred on the machined inner surface of hole.

Indicating potential turbulent flow conditions for applying *T* = ~313.15 °K and *p* = 8 MPa is possible during the drilling process, but whirls accompanying the turbulent flow can hinder sufficient working fluid flow via the gap area and the side gap near the hole bottom. During the experiment 2 the properties of deionized water such as density and viscosity were reduced by using the higher deionized water temperature, but during the conditions did not influence the improvement removing of debris from the gap area. Hence, the presence of whirls could be possibility.

The results analysis shows that the properties of deionized water influence significantly the thermal phenomena present in the interelectrode area. The obtained higher electrical conductivity of deionized water by use of the higher initial temperature (*T* = ~313.15 °K), enabled removing the material in similar ranges of both processes (ECM and EDM) during single pulse time. Further studies should focus on more detailed analysis impact of the deionized water properties on the EDD process and also the influence of the physical-mechanical properties of both electrode materials.

## 5. Conclusions

The use of electrical discharge machining with the application of deionized water to drill high aspect ratio holes in the Inconel 718 alloy demonstrates a significant potential. One of the advantages of the application of this kind of working fluid is the possibility of removing material by simultaneous electrochemical dissolution and electrical discharges in a single pulse. Where deep holes are made using the EDD process, an important factor is effective removal of erosion products from the gap area (such as eroded particles and gas bubbles). Insufficient flushing of the gap area contributes to decreasing the dimensional and shape accuracy (aspect ratio, conicity), the inner surface homogeneity of the holes, and the process performance (drilling speed, linear tool wear). In the experimental research conducted, the change in the properties of the working fluid under the influence of temperature and the working fluid pressure affect the nature of the fluid flow through the machining area. The analysis of the results enables the formulation of the following conclusions:

1. The EDD experimental research with the use of deionized water (with given the initial temperature ~313.15 °K and the initial pressure 8 MPa) in the Inconel 718 alloy confirmed the possibility of making high aspect ratio holes (*AR* > 30) and decreasing the side gap thickness by about 40%.
2. For drilling high aspect ratio through holes, the optimum machining parameters were pulse time *t_i_* = 282 µs, discharge voltage amplitude *U* = 120 V, current amplitude *I* = 4.32 A and pulse of time *t_off_* = 500 µs.
3. The setting of a higher initial working-fluid temperature (~313.15 °K) increased the electrical conductivity of deionized water, which reinforced the electrochemical reactions in a single pulse. As a result, in the diameter of the hole top, there were no burrs or heat affected zones. The homogeneity of the inner surface of the hole was also improved (without micro-cracks and erosion micro-craters).
4. On the surface around the hole top, the material is seriously damaged as a result of excessive electrochemical dissolution. The presence of oxygen was detected in cavities and darker zones between the cavities. This indicates oxidation of the machined surface and the creation of a passive layer.
5. The bottom diameter is too large and larger than the top diameter by about 40%. This is due to the accumulation of debris at the hole bottom and the occurrence of secondary discharges. Removal of eroded material from the hole bottom might have been made difficult by the whirls resulting from a turbulent fluid flow.
6. The dark oval traces on the electrode surface and the oval shape of the cavities on the inner surface of the hole can indicate the occurrence of electrical discharges on the small areas between the gas bubbles.
7. The complex nature of the phenomena within the gap area during the EDD process with the use of deionized water requires further analysis and better understanding. Further experimental research should focus on the impact of the physical and mechanical properties of the Inconel 718 alloy and tool electrode material on the process.

## Figures and Tables

**Table 1:** Chemical composition of Inconel 718 (wt. %).

Ni	Cr	Fe	Nb	Mo	Ti	Al	Co	Mn	C	Si	P
50.0–55.0	17.0–21.0	Balance	4.75–5.5	2.8–3.3	0.65–1.15	0.2–0.8	<1.0	<0.35	<0.08	<0.35	<0.015

**Table 2:** Physical and mechanical properties of the workpiece and of the electrode material, for *T* = 298.15 °K and *T* = 291.15 °K, respectively [51,52,53,54].

Property	Workpiece	Tool
Density, (kg/m^3^)	8190	8960
Heat capacity, (J/(kg °K))	435	385
Thermal conductivity, (W/(m °K))	8.9	388
Melting temperature range, (°K)	1533–1609	1338–1356

**Table 3:** Machining parameters.

Input Parameters	Output Parameters
Pulse time, *t_i_* (μs)	Drilling speed, *v* (μm/s)
Current amplitude, *I* (A)	Linear tool wear, *TW* (%)
Discharge voltage amplitude, *U* (V)	Taper angle, *tap_α_*
	Aspect ratio hole, *AR*
	Side gap thickness, *S_b_* (µm)

**Table 4:** Process parameters and their levels.

Coded Parameter	Real Parameter	Level				
		1	2			3
*X_1_*	*U* (V)	80	100			120
		1	2	3	4	5
*X_2_*	*t_i_* (µs)	100	282	550	818	999
*X_3_*	*I* (A)	3	3.33	3.83	4.32	4.65

**Table 5:** Research plan and the results of the experiments.

Experiment No.	*X_1_*	*X_2_*	*X_3_*	*U* (V)	*t_i_* (µs)	*I* (A)	*v* (μm/s)	*TW* (%)	*tap_α_*	*AR*	*S_b_* (µm)
1	1	2	2	80	282	3.33	6.69	39.27	−0.00313	25	102
2	1	2	4	80	282	4.32	8.89	51.58	−0.00446	28	128
3	1	4	2	80	818	3.33	5.92	54.95	−0.00247	22	122
4	1	4	4	80	818	4.32	7.90	81.54	−0.00593	26	121
5	3	2	2	120	282	3.33	8.79	45.68	−0.00767	29	120
6	3	2	4	120	282	4.32	12.56	52.70	−0.00903	27	130
7	3	4	2	120	818	3.33	6.62	22.86	−0.0026	24	127
8	3	4	4	120	818	4.32	9.33	73.55	−0.00933	26	129
9	1	3	3	80	550	3.83	7.81	47.30	−0.0036	27	103
10	3	3	3	120	550	3.83	9.06	49.61	−0.00409	31	120
11	2	1	3	100	100	3.83	8.65	39.46	−0.00872	29	89
12	2	5	3	100	999	3.83	7.55	72.08	−0.00424	27	119
13	2	3	1	100	550	3.00	7.37	33.99	−0.00123	31	111
14	2	3	5	100	550	4.65	12.27	51.44	−0.01208	25	139
15	2	3	3	100	550	3.83	7.87	48.90	−0.00456	29	94
16	2	3	3	100	550	3.83	7.94	50.72	−0.00436	29	96
17	2	3	3	100	550	3.83	8.06	55.09	−0.00227	30	107
18	2	3	3	100	550	3.83	8.76	55.10	−0.00188	30	111
19	2	3	3	100	550	3.83	8.43	56.07	−0.00521	29	115
20	2	3	3	100	550	3.83	8.16	53.70	−0.00215	29	112

**Table 6:** Selected physical properties of the deionized water during saturation pressure.

Physical Properties	Working Fluid Temperature, *~T* (°K)			
	298.15	303.15	313.15	373.15
Density, (kg/m^3^)	997.1	995.7	992.2	958.4
Specific heat, (kJ/(kg°K))	4.178	4.176	4.175	4.211
Thermal conductivity, (W/(m°K))	0.606	0.615	0.633	0.682
Dynamic viscosity, (kg/(s∙m))∙10^6^	880.637	792.377	658.026	277.528
Kinematic viscosity, (m^2^/s)∙10^6^	0.896	0.804	0.661	0.296
Electrical conductivity, (µS/cm)	0.05501	0.07101	0.11351	0.79303
Resistivity, (MΩcm)	18.180	14.082	8.810	1.261

**Table 7:** Process parameters and their levels.

Coded Factor	Real Parameter	Level		
		1	2	3
*X*_11_	Initial working-fluid temperature, *~T* (°K)	298.15	303.15	313.15
*X*_22_	Initial working-fluid pressure, *p* (MPa)	5	7	8

**Table 8:** Research plan and results of the experiments; * *Re* > 2300 turbulent flow is considered.

Experiment No.	Coded Factor		Real Parameter		Response Variables		
	*X_11_*	*X_22_*	*~T* (°K)	*p* (MPa)	*Q* (m^3^/s)∙10^7^	*v_f_* (m/s)	*Re*
1	1	1	298.15	5	4.778	13.22	1582
2	3	1	313.15	5	4.333	11.99	1945
3	1	3	298.15	8	6.667	18.44	2208
4	3	3	313.15	8	5.833	16.14	2619*
5	1	2	298.15	7	5.444	15.06	1803
6	3	2	313.15	7	5.417	14.98	2431*
7	2	1	303.15	5	4.722	13.06	1743
8	2	3	303.15	8	6.278	17.37	2317*
9	2	2	303.15	7	5.667	15.67	2091
10	2	2	303.15	7	5.708	15.79	2107
11	2	2	303.15	7	5.817	16.09	2147

**Table 9:** Diagnostic statistics for the regression.

	*Q* (*T*, *p*)	*Re* (*T*, *p*)
*R^2^*	0.9593	0.9761
*R^2^* adjusted	0.9187	0.9521
*p*-Value	0.0017	0.00047

## Supplementary Materials

The following are available online https://www.mdpi.com/1996-1944/13/6/1476/s1. Supplementary section S1 presenting the ANOVA analysis for: drilling speed *v*, linear tool wear *TW*, the taper angle *tap_α_*, the side gap thickness *S_b_* and the aspect ratio hole *AR*.
