# Supplementary material for: Impact of the Deionized Water on Making High Aspect Ratio Holes in the Inconel 718 Alloy with the Use of Electrical Discharge Drilling
Source: Materials (Basel). 2020 Mar 24;13(6):1476. doi: 10.3390/ma13061476 (PMC7143403; doi:10.3390/ma13061476)
Supplement: Supplementary file 1 [file materials-13-01476-s001.pdf]

materials

Supplementary Materials

Impact of the Deionized Water on Making High Aspect Ratio Holes in the Inconel 718 Alloy with the Use of Electrical Discharge Drilling

Magdalena Machno [1,*], Rafał Bogucki [2], Maciej Szkoda [1] and Wojciech Bizoń [3]

1 Institute of Rail Vehicles, Faculty of Mechanical, Cracow University of Technology, 31-155 Cracow, Poland; maciej.szkoda@pk.edu.pl.
2 Institute of Materials Engineering, Faculty of Materials Engineering and Physics, Cracow University of Technology, 31-155 Cracow, Poland; rbogucki@mech.pk.edu.pl.
3 Institute of Production Engineering, Faculty of Mechanical, Cracow University of Technology, 31-155 Cracow, Poland; wojciech.bizon@pk.edu.pl.
* Correspondence: magdalena.machno@pk.edu.pl.; Tel.: +48-12-374-36-56

Received: 5 February 2020; Accepted: 20 March 2020; Published: date

Supplementary Section S1

The results of the ANOVA analysis for drilling speed *v* and linear tool wear *TW* are given in Table 1 (where: *DF* is degrees of freedom, *Adj SS* is the adjusted sums of squares, and *Adj MS* is the adjusted means squares). The drilling parameters effecting the process performance at the applied significance level of $\alpha = 0.05$ have the "*" sign. Additionally, for *TW* the selected relevant parameter is the discharge voltage *U*.

Table 1. ANOVA for drilling speed *v* and linear tool wear *TW*.

	v					*TW*				
**Source**	***Adj SS***	***DF***	***Adj MS***	***F* - Value**	***p* - Value**	***Adj SS***	***DF***	***Adj MS***	***F* - Value**	***p* - Value**
U	9.543	1	9.544	35.380	0.000216*	105.685	1	105.685	2.2171	0.170674*
U^2	0.260	1	0.260	0.962	0.352311	11.482	1	11.482	0.241	0.635325
ti	6.627	1	6.627	24.568	0.000784*	762.634	1	762.634	15.999	0.003111*
ti^2	0.213	1	0.213	0.790	0.397362	73.623	1	73.623	1.545	0.245357
I	28.486	1	28.486	105.604	0.000003*	1221.701	1	1221.701	25.629	0.000679*
I^2	2.908	1	2.907	10.778	0.009485*	57.490	1	57.490	1.206	0.300630
$U \cdot ti$	2.075	1	2.075	7.693	0.021626*	303.887	1	303.887	6.375	0.032509*
$U \cdot I$	0.880	1	0.880	3.262	0.104366	45.149	1	45.149	0.947	0.355879
$ti \cdot I$	0.305	1	0.305	1.132	0.315151	483.421	1	483.421	10.141	0.011104*
Residual Error	2.428	9	0.270	-	-	429.023	9	47.669	-	-
Total	79.145	18	-	-	-	3326.029	18	-	-	-

The results of the ANOVA analysis for the taper angle tap_α and the side gap thickness S_b are presented in Table 2. The process parameters which influence the taper angle and the side gap thickness at the applied significance level of $\alpha = 0.05$ have the "*" sign. Also, for the tap_α the discharge voltage is regarded as significant parameter, but for the S_b the discharge voltage and the currentamplitude.

materials MDPI

Table 2. ANOVA for the taper angle tap_{α} and the side gap thickness S_b.

Source	*Tapα*					*Sb*				
	Adj SS	*DF*	*Adj MS*	*F*-Value	*p*-Value	*Adj SS*	*DF*	*Adj MS*	*F*-Value	*p*-Value
U	0.000005	1	0.000005	2.786	0.129449*	116.705	1	116.705	1.632	0.233394*
U^2	0.000001	1	0.000001	0.393	0.546172	1264.706	1	1264.706	17.686	0.002288*
ti	0.000018	1	0.000018	10.436	0.010314*	653.773	1	653.773	9.142	0.014394*
ti^2	0.000012	1	0.000012	7.259	0.024627*	0.269	1	0.269	0.004	0.952431
I	0.000044	1	0.000044	25.709	0.000671*	9.980	1	9.980	0.140	0.717367*
*I*2	0.000002	1	0.000002	1.138	0.313799	116.906	1	116.906	1.635	0.233023
$U \cdot ti$	0.000003	1	0.000003	1.644	0.231775	65.029	1	65.029	0.909	0.365193
$U \cdot I$	0.000000	1	0.000000	0.191	0.672401	19.105	1	19.105	0.267	0.617698
$ti \cdot I$	0.000018	1	0.000018	10.743	0.009566*	627.329	1	627.329	8.773	0.015912*
Residual Error	0.000015	9	0.000002	-	-	643.593	9	71.510	-	-
Total	0.000133	18	-	-	-	3510.287	18	-	-	-

The results of the ANOVA analysis for the aspect ratio hole *AR* is presented in Table 3. The process parameters which impact of the aspect ratio hole at the applied significance level of $\alpha = 0.05$ have the "*" sign. Additionally, the process parameters such as the discharge voltage, the pulse time and the current amplitude are regarded as a significant parameter.

Table 3. ANOVA for the aspect ratio hole *AR*.

Source	Adj SS	DF	Adj MS	F-Value	p-Value
U	10.074	1	10.074	3.020	0.116235*
U^2	18.874	1	18.875	5.659	0.041307*
ti	11.450	1	11.450	3.433	0.096916*
ti^2	18.104	1	18.104	5.428	0.044765*
I	10.486	1	10.486	3.144	0.109963*
I^2	0.353	1	0.353	0.106	0.752351
$U \cdot ti$	0.040	1	0.040	0.012	0.915292
$U \cdot I$	2.270	1	2.270	0.681	0.430691
$ti \cdot I$	0.914	1	0.914	0.274	0.613246
Residual Error	30.018	9	3.335	-	-
Total	99.938	18	-	-	-

© 2020 by the authors. Submitted for possible open access publication under the terms and conditions of the Creative Commons Attribution (CC BY) license (http://creativecommons.org/licenses/by/4.0/).
